# Ownership and use of long-lasting insecticidal nets three months after a mass distribution campaign in Uganda, 2021

**DOI:** 10.1186/s12936-022-04401-5

**Published:** 2022-12-03

**Authors:** Andrew Kwiringira, Carol Nanziri, Edirisa Juniour Nsubuga, Stella Martha Migamba, Vivian Ntono, Immaculate Atuhaire, Sherry Rita Ahirirwe, Alice Asio, Shaban Senyange, Petranilla Nakamya, Veronicah Masanja, Sarah Elayeete, Allan Komakech, Hildah T. Nansikombi, Patience Mwine, Rose Nampeera, Alex Ndyabakira, Paul Okello, Richard Migisha, Lilian Bulage, Benon Kwesiga, Daniel Kadobera, Damian Rutazaana, Julie R. Harris, Alex R. Ario

**Affiliations:** 1 Uganda Public Health Fellowship Program, Kampala, Uganda; 2 Uganda National Institute of Public Health, Kampala, Uganda; 3 Uganda National Malaria Control Program, Ministry of Health, Kampala, Uganda; 4 US Centers for Disease Control and Prevention, Kampala, Uganda

**Keywords:** Malaria, Long-lasting insecticidal nets, Ownership and use, Mass campaigns, Uganda

## Abstract

**Background:**

Uganda conducted its third mass long-lasting insecticidal net **(**LLIN) distribution campaign in 2021. The target of the campaign was to ensure that 100% of households own at least one LLIN per two persons and to achieve 85% use of distributed LLINs. LLIN ownership, use and associated factors were assessed 3 months after the campaign.

**Methods:**

A cross-sectional household survey was conducted in 14 districts from 13 to 30 April, 2021. Households were selected using multistage sampling. Each was asked about LLIN ownership, use, duration since received to the time of interview, and the presence of LLINs was visually verified. Outcomes were having at least one LLIN per two household members, and individual LLIN use. Modified Poisson regression was used to assess associations between exposures and outcomes.

**Results:**

In total, 5529 households with 27,585 residents and 15,426 LLINs were included in the analysis. Overall, 95% of households owned ≥ 1 LLIN, 92% of the households owned ≥ 1 LLIN < 3 months old, 64% of households owned ≥ 1 LLIN per two persons in the household. Eighty-seven per cent could sleep under an LLIN if every LLIN in the household were used by two people, but only 69% slept under an LLIN the night before the survey. Factors associated with LLIN ownership included believing that LLINs are protective against malaria (aPR = 1.13; 95% CI  1.04–1.24). Reported use of mosquito repellents was negatively associated with ownership of LLINs (aPR = 0.96; 95% CI 0.95–0.98). The prevalence of LLIN use was 9% higher among persons who had LLINs 3–12 months old (aPR = 1.09; 95% CI  1.06–1.11) and 10% higher among those who had LLINs 13–24 months old (aPR = 1.10; 95% CI  1.06–1.14) than those who had LLINs < 3 months old. Of 3,859 LLINs identified in the households but not used for sleeping the previous night, 3250 (84%) were < 3 months old. Among these 3250, 41% were not used because owners were using old LLINs; 16% were not used because of lack of space for hanging them; 11% were not used because of fear of chemicals in the net; 5% were not used because of dislike of the smell of the nets; and, 27% were not used for other reasons.

**Conclusion:**

The substantial difference between the population that had access to LLINs and the population that slept under LLINs indicates that the National Malaria Control Programme (NMCP) may need to focus on addressing the main drivers or barriers to LLIN use. NMCP and/or other stakeholders could consider designing and conducting targeted behaviour change communication during subsequent mass distribution of LLINs after the mass distribution campaign to counter misconceptions about new LLINs.

## Background

Over the past 20 years, the scale-up of malaria control efforts has led to marked reductions in morbidity and mortality globally [1, 2]. An estimated 663 million malaria cases were averted by malaria control interventions between 2000 and 2015; nearly 70% of cases averted were attributed to the use of long-lasting insecticidal nets (LLINs) [1]. However, global progress has slowed in recent years, particularly in sub-Saharan Africa, which accounted for 94% of the world’s 219 million cases in 2019 [2]. Uganda has the third highest global burden of malaria cases (5%) and the eighth highest level of malaria deaths (3%).

LLINs are one of the core interventions recommended by the World Health Organization (WHO) to reduce malaria transmission and prevent malaria in high-risk communities [2]. LLINs have been shown to reduce malaria incidence among children under 5 years and pregnant women by up to 50% and all-cause mortality in children by 20% [3]. Since 2013, the government of Uganda has conducted three mass LLIN distribution campaigns to achieve universal LLIN coverage (have at least one LLIN for two persons in the household for 100% of households) and reduce inequality in the ownership of LLINs between poor and wealthy households. The most recent mass distribution campaign occurred in 2020/21, when 27 million LLINs were distributed nationwide [4].

Despite LLIN distribution campaigns, the malaria burden remains high in Uganda. Malaria accounts for 30–50% of outpatient visits at health facilities and 15–20% of all hospital admissions in the country [5]. The Malaria Indicator Survey conducted in Uganda in 2018/19 showed that 54% of households own at least one LLIN for two people and 59% of the population used the LLINs while sleeping [6]. Studies that have documented barriers to LLIN use have shown lack of sufficient space to hang the net, lack of enough nets for a household, discomfort with the net material, age of the LLIN, and belief that there are harmful chemicals in new LLINs [7]; however, different settings have unique and dynamic barriers to LLIN use and may require unique strategies [8]. This survey was conducted 3 months after the 2020/2021 mass LLIN distribution campaign to estimate ownership and use and identify barriers to LLIN use in 14 districts in Uganda.

## Methods

### Study design and setting

**Fig. 1:**
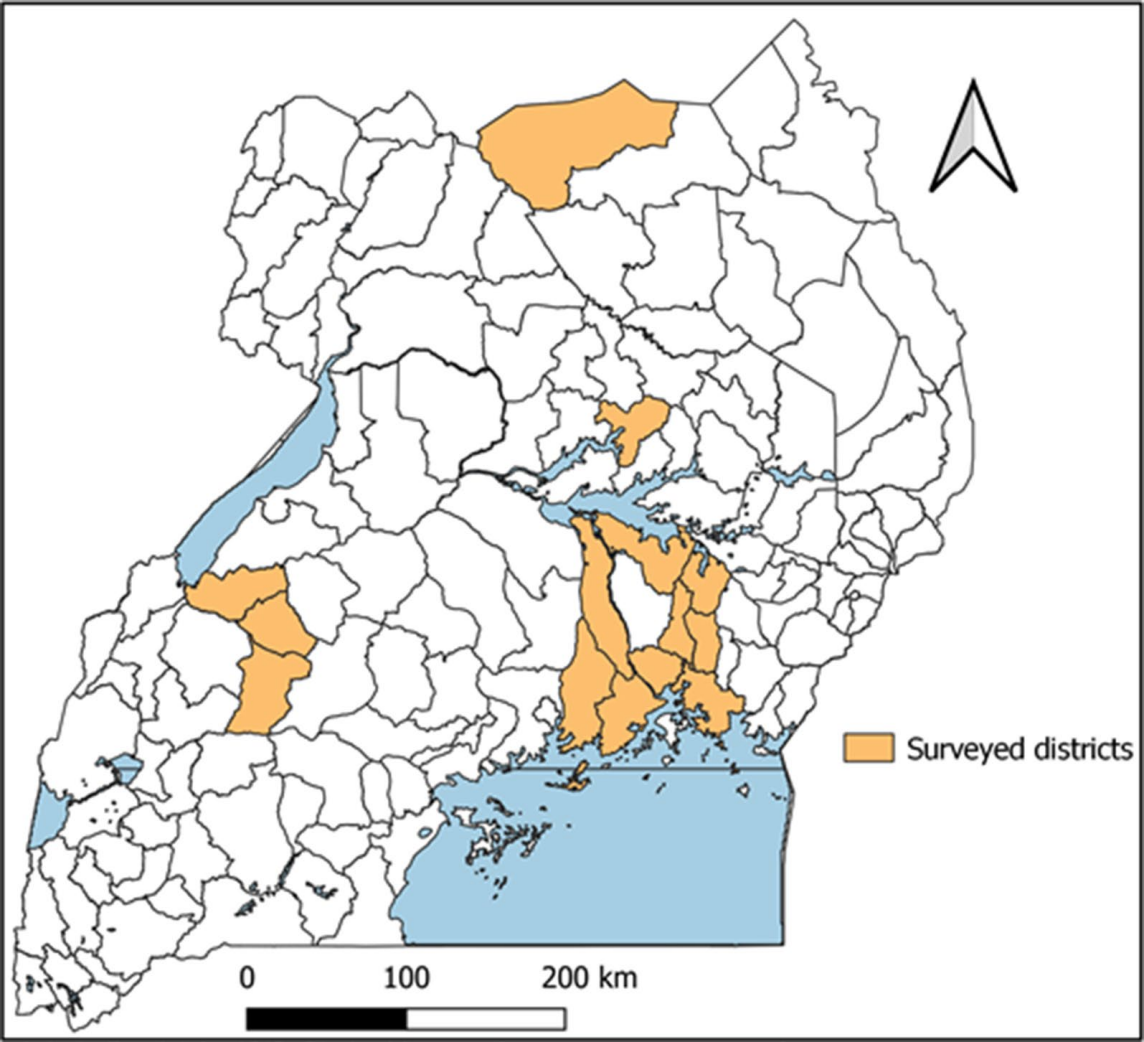
Location of the 14 districts surveyed three months after a mass distribution campaign, Uganda, 2021

A cross-sectional household survey was conducted in 14 districts (Buikwe, Buyende, Dokolo, Iganga, Jinja, Kagadi, Kaliro, Kayunga, Kibaale, Kyegegwa, Lamwo, Luuka, Mayuge, Mukono) in Uganda between 13 and 30 April 2021 (Fig. 1). These districts were chosen because they received LLINs in the last phase of the mass distribution campaign in January 2021, three months before the survey.

### Sample size and sampling

The sample size was calculated for precision based on an estimated 84% of households having at least one LLIN [6], 95% confidence, an error of +/− 5%, and a design effect of 2, for a total of 412 households per district. The mass distribution of LLINs was conducted across the country in phases between August 2020 and March 2021. Fourteen districts were selected that were targeted in the last phase of the campaign. From each district, one sub-county, one parish from each of the selected sub-counties, and two villages from the selected parish were randomly selected using a random number generator. Probability proportional to size sampling was used to determine the number of households to sample from the selected villages and a systematic sampling process was used from a list of households in each village to select households for the survey.

### Study outcomes, dependent variables and data collection

The Roll Back Malaria Monitoring and Evaluation Reference Group (RBM MERG) indicators were used to report LLIN ownership and use [9]. The primary outcomes were the percentage of households with at least one LLIN, percentage of surveyed households that had at least one LLIN for every two persons who stayed in the household the previous night (the number of households that had a people to LLIN ratio of 2.0 or less divided by the total number of surveyed households), percentage of population with access to an LLIN in their household (the number of household members who could sleep under an LLIN if each LLIN in the household were used by two people, divided by the total number of individuals who had spent the previous night in surveyed households), percentage of population that slept under an LLIN the previous night (the number of individuals who slept under an LLIN the previous night divided by the total number of individuals who spent the previous night in surveyed households), and percentage of existing LLINs used the previous night (number of LLINs in surveyed households that were used by anyone the previous night divided by the number of LLINs in the surveyed households). Further identified in households were LLINs not being slept under and assessed why they were not being used.

Households were visited and the head of household or one of his or her adult dependants was interviewed. If no appropriate respondent was found at the house, another visist was scheduled another later that day. At least three attempts were made to reach a respondent before dropping the household without replacing it. The household questionnaire included a household member roster, questions about the mosquito net(s) owned by households, and participants’ beliefs about LLINs. For each net identified in the household, the brand was confirmed and questions were asked about the source of the LLIN and which member(s) in the household used the net previous night; these data were input into the household member database. The duration from the time LLINs were received until the time of interview was categorized as < 3 months, 3–12 months, > 12–24 months, > 24 months and unknown (Table 1).

Self-reported duration was corroborated with records of village health teams during the interview. LLINs were examined for material type (polyester vs. polyethylene).

### Data management and statistical analysis

Data was collected using hand-held Android phones that were programmed to include range checks and internal consistency checks. Data were transferred daily to a secure server on a private network at the data core facility at Ministry of Health, Kampala. All statistical analyses were carried out using STATA version 14 (Statcorp, College Station, TX, USA). Household and household member characteristics, estimation of LLIN ownership and use are presented as percentages. Socio-economic status (SES) of each participating household was calculated by creating a wealth index based on materials used to construct the house, household amenities and assets owned. A weighted score for each household was calculated using principal component analysis (PCA) and divided households into SES indices [10]. Sampling weights were calculated to account for clustering by district, sub-county and parish. Bivariate analysis was conducted between each of the outcomes (household ownership of at least one LLIN and use of any LLIN) and the independent variables. A multivariate analysis was conducted using modified Poisson regression and the measure of association was prevalence ratios (PRs) and 95% confidence intervals. The modified Poisson regression model was used to avoid under estimation of standard errors for the estimated prevalence ratios [11]. Prevalence of both LLIN ownership and LLIN use was more than 10%. P-values of < 0.05 showed statistically significant associations between the outcomes and the independent variables. Independent variables with P-values ≤ 0.1 in bivariate analysis were considered for inclusion in the multivariable model.

## Results

### Household and household member characteristics

A total of 5529 households and 27,584 household members were included in the survey. 5529 households out of the expected 5768 were surveyed and the response rate was evenly distributed between the districts; 3–4% of the expected households were not surveyed in each of the surveyed districts. Mean household size was five persons (range, 1–25); 4220 (15%) household members were < 5 years of age. A total of 15,426 nets were found in these households. Of these, 12,260 (80%) nets were distributed in 2020/21 through the government mass distribution mechanism.

### Long-lasting insecticidal nets ownership

Overall, 96% (95% CI 94–99%) households owned at least one LLIN and 64% (95% CI 59–72) households had at least one LLIN for every two persons in the household (targeted coverage per household). Most (4976; 92%) households had at least one LLIN that was < 3 months old.

### Long-lasting insecticidal net use

Among 27,434 household members, 23,977 (87%) could sleep under an LLIN if every LLIN in the household were used by two people, and 18,954 (69%) slept under an LLIN the night before the survey. Overall, 11,466 (74%) of 15,426 existing LLINs in the households were used the night before the survey. Of 3859 LLINs not used during sleeping the previous night, 3250 (84%) were < 3 months old. Among these, 1333 (41%) were not used because owners were using old LLINs; 358 (11%) were not used because of fear of chemicals in the net.

### Factors associated with household ownership of at least one LLIN

Household LLIN ownership (having any LLIN in a household) was slightly higher among households with a high wealth index compared to households with a low wealth index (aPR = 1.02; 95% CI   1.01–1.04), and slightly lower among households in which respondents reported using mosquito repellents compared to those in which they reported not using repellents (aPR = 0.96; 95% CI  0.95–0.98) (Table 2). The prevalence of household LLIN ownership was 13% higher among households where respondents believed LLIN would protect them from malaria compared to households where respondents did not believe LLINs would protect them from malaria (aPR = 1.13; 95% CI  1.04–1.24) (Table 2).

**Table 1:** Characteristics of long-lasting insecticidal nets, 3 months after a mass distribution campaign, Uganda, 2021 (n = 15,426 LLINs)

Characteristic variable	Frequency (n)	Percentage
LLIN texture		
Polyester	6,189	40.1
Polyethylene	2,542	15.4
Polyester and polyethylene	4,866	31.5
Not sure	1,829	13.0
LLIN source		
Mass distribution 2020/21	12,260	79.5
Mass distribution 2017	2,201	14.3
Antenatal clinic	505	3.3
Self-purchased	271	1.8
Others	90	0.6
Unknown	99	0.6
LLIN age		
New (< 3 months)	11,101	71.9
3–12 months	1,583	10.3
> 12–24 months	398	2.6
> 24 months	2,195	14.2
Unknown	149	1.0

**Table 2:** Factors associated with household long-lasting insecticidal nets ownership, 3 months after a mass distribution campaign, Uganda, 2021

Variable	LLIN ownership		PR	95% CI	p	aPR	95% CI	P
	Yes	No						
Wealth index								
Low	1805	98	1.00			1.00		
Medium	1704	71	1.01	0.99–1.02	0.10	1.01	0.99–1.03	0.06
High	1769	65	1.01	1.00–1.03	0.02	1.02	1.01–1.04	0.001
Repellent use								
No	4345	161	1.00			1.00		
Yes	947	75	0.96	0.94–0.98	< 0.001	0.96	0.95–0.98	< 0.001
Nets protect from malaria								
No	76	13	1.00			1.00		
Yes	5,125	174	1.13	1.03–1.23	0.01	1.13	1.04–1.24	0.004
Not sure	92	49	0.76	0.66–0.89	< 0.001	0.77	0.66–0.89	< 0.001
Malaria is a serious condition								
No	119	18	1.00					
Yes	5174	218	1.10	1.03–1.18	0.003			

* PR* prevalence ratio, * aPR* adjusted prevalence ratio, * CI* confidence interval

### Factors associated with long-lasting insecticidal nets used

Compared to LLINs < 3 months old (i.e., newly-distributed LLINs), the prevalence of LLIN use was higher both for LLINs 3–12 months old (aPR = 1.09; 95% CI 1.06–1.11) and LLINs 13–24 months old (aPR = 1.10; 95% CI  1.06–1.14) (Table 3). The use of LLINs with polyester material was 4% lower than use of LLINs with polyethylene material (aPR = 0.96; 95% CI   0.94–0.97) (Table 3). Participants who reported that LLINs were hung on their bed or sleeping space were more likely to use the net compared to those who reported that nets were not hung (aPR = 6.29; 95% CI  5.83–6.78) (Table 3).

**Table 3:** Factors associated with long-lasting insecticidal nets used, 3 months after a mass distribution campaign, Uganda, 2021

Variable	LLIN used the night before the survey		PR	95% CI	p	aPR	95% CI	P
	Yes	No						
Age of net (months)								
< 3	7814	3250	1.00			1.00		
3–12	1428	153	1.27	1.25–1.30	< 0.001	1.09	1.06–1.11	< 0.001
13–24	364	34	1.29	1.25–1.34	< 0.001	1.10	1.06–1.14	< 0.001
> 24	1797	387	1.17	1.14–1.19	< 0.001	1.02	0.99–1.05	0.17
Unknown	63	35	0.91	0.78–1.06	0.21	1.06	0.98–1.15	0.15
Net texture								
Polyethylene	2150	387	1.00			1.00		
Polyester	4658	1521	0.89	0.87–0.91	< 0.001	0.96	0.94–0.97	< 0.001
Polyester and polyethylene	3 283	1564	0.79	0.78–0.82	< 0.001	0.97	0.95–0.98	< 0.001
Not sure	1375	387	0.92	0.89–0.95	< 0.001	0.92	0.89–0.95	< 0.001
Source of net								
2017 mass distribution	1827	365	1.00			1.00		
2021 mass distribution	8843	3360	0.87	0.85–0.89	< 0.001	0.98	0.95–1.01	0.14
Self-purchased	233	38	1.03	0.98–1.09	0.24	0.97	0.94–1.01	0.17
Antenatal clinic	450	55	1.07	1.03–1.11	< 0.001	0.98	0.96–1.01	0.25
Other	113	41	0.88	0.79–0.97	0.01	0.92	0.86–0.99	0.02
Net hanging over bed								
No	614	3468	1.00			1.00		
Yes	10,852	391	6.42	5.97–6.90	< 0.001	6.29	5.83–6.78	< 0.001
Net condition								
No holes	9563	3476	1.00					
One or few holes	1328	119	1.25	1.23–1.27	< 0.001			
Many holes	553	224	0.97	0.93–1.02	0.20			
Unknown	22	40	0.48	0.35–0.68	< 0.001			

* PR* prevalence ratio, * aPR* adjusted prevalence ratio, * CI* confidence interval

## Discussion

Three months after a mass LLIN distribution campaign in Uganda, nearly all households owned at least one LLIN, and six in 10 households owned the targeted number of LLINs (at least one LLIN per two persons in the household). More than eight in 10 residents could sleep under an LLIN if every LLIN in the household were used by two people. However, only two-thirds of residents slept under an LLIN the previous night. LLIN ownership was associated with belief in their protectiveness against malaria, and LLIN use was associated with net age.

Currently, the targets in the NMCP strategic plan for households with at least one LLIN and proportion of people sleeping under LLIN were set at 80% [4]. The WHO also calls for procuring LLINs with the goal of providing each household with one LLIN for two persons [2]. These expectations could create a feeling of failure since 64% households had at least one LLIN for two people, falling below the target. However, recent literature shows that a target of 80% for households owning at least one LLIN for two people is not achievable at a national or even sub-national level [12]. The proportion of the population with access to an LLIN within the household is the key indicator of universal coverage [12]. This study showed that more than eight in 10 residents could sleep under an LLIN if every LLIN in the household were used by two people.

Beyond achieving universal coverage, a related metric of success after a mass distribution campaign is the proportion of household members sleeping under the LLINs [4]. This study showed an increase in the proportion of the population that slept under an LLIN the previous night from 59%, reported in UMIS 2018/19, to 69% after the 2021 mass distribution campaign. However, this achievement also falls short of the NMCP target of having 85% of the population sleeping under an LLIN [13]. Some reasons that people may not use nets, even when they are provided, include lack of sufficient space to hang the net, discomfort with the net material, belief that there are harmful chemicals in new LLINs, and a desire to save LLINs for use when a household member is pregnant [3, 7]. This study showed that older nets were more likely to be used than the newest nets, the preference for polyester LLINs was slightly lower than that for polyethylene LLINs, and there was an increased use of LLINs among people who believed that they were protective against malaria. There is evidence that behavioural change communication (BCC), either through mass media [14], intensive and repeated inter-personal communication, or material incentives [15] can promote changes in behaviour, beliefs and attitudes towards LLINs [16]. While BCC through mass media is the main approach used in Uganda [4], more data are needed to identify the optimal mix of approaches to maximize LLIN use after mass distribution campaigns.

This study showed that inequality in LLIN ownership between households with low and high wealth indexes was minimal. The minimal inequality observed in this study could be due to improved coverage on LLINs. However, this analysis is not based on randomly selected sample for the household and some districts may be more affluent than others, this could potentially lead to bias in these results. A study evaluated the change in equity in ownership of LLINs in 19 sub-Saharan African countries and concluded that equity of net ownership had improved in 13 countries, including Uganda, after mass distribution of LLINs [17]. The ownership of at least one LLIN was lower among households where respondents reported using mosquito repellents, compared with those that did not use repellents. Respondents who had repellents may have believed that repellents were protective enough and they did not need LLINs; however, it is also possible that people who did not receive or have enough LLINs may have used repellents as an alternative. While mosquito repellents do provide protection against malaria infection [18, 19], the combined use of mosquito repellent during evening outdoor activities followed by the use of LLINs during bedtime at community level significantly reduces malaria infection compared with repellent use alone [20]. Both education and BCC may be required during LLIN distribution to ensure that repellents are used as adjuvants, not substitutes, for LLINs.

This study has some limitations. First, LLIN use was self-reported, which could have underestimated or overestimated the actual use of LLINs. Second, reported use of LLINs the night before the survey only captures use at one point in time and might not represent regular use. Although this is the recommended approach to measuring LLIN use [21], a meta-analysis showed that self-reported measures overestimate LLIN adherence by 13% relative to objective measures [22], suggesting that the true proportion of the population who slept under LLIN the previous night could be lower than what this study estimated. Third, ability to understand why individuals chose to use nets or not is limited by the quantitative nature of the questionnaire. Further exploration using qualitative research methods would be required to better understand local perceptions and why they are hesitant to take up new LLINs. Fourth, the overall sample for the survey only included one sub-county, one parish, and two villages per district, which may not be representative of the district as a whole. If these sampling units in the district are not homogeneous, this could potentially lead to bias in any direction in results. This approach was employed because of limited resources and to get the broadest sample possible geographically.

## Conclusion

LLIN use fell well short of the national target immediately after distribution of LLINs in Uganda. The substantial difference between the population that had access to LLINs and the population that used the LLINs previous night before the survey indicates that the NMCP may need to focus on addressing the main drivers or barriers to LLIN use. NMCP and/or other stakeholders could consider designing and conducting targeted BCC during subsequent mass distribution of LLINs after the mass distribution campaign to counter misconceptions about new LLINs.

## Data Availability

The datasets used and/or analysed for the current study are available from the corresponding author on reasonable request.

## References

[1] Bhatt S, Weiss D, Cameron E, Bisanzio D, Mappin B, Dalrymple U (2015). The effect of malaria control on *Plasmodium falciparum* in Africa between 2000 and 2015. Nature.

[2] WHO. World malaria report 2020. Geneva, World Health Organization; 2020.

[3] Alaii JA, Van Den Borne H, Kachur SP, Shelley K, Mwenesi H, Vulule JM (2003). Community reactions to the introduction of permethrin-treated bed nets for malaria control during a randomized controlled trial in western Kenya. Am J Trop Med Hyg..

[4] Ministry of Health. Uganda malaria reduction strategic plan. Kampala, Uganda; 2014–2020.

[5] Ministry of Health (2018). Annual health sector performance report.

[6] Ministry of Health (2018). Uganda malaria indicator survey 2018–2019.

[7] Baume CA, Reithinger R, Woldehanna S (2009). Factors associated with use and non-use of mosquito nets owned in Oromia and Amhara regional states, Ethiopia. Malar J.

[8] Ntuku HM, Ruckstuhl L, Julo-Réminiac J-E, Umesumbu SE, Bokota A, Tshefu AK (2017). Long-lasting insecticidal net (LLIN) ownership, use and cost of implementation after a mass distribution campaign in Kasaï Occidental Province, Democratic Republic of Congo. Malar J.

[9] Evaluation MEASURE. MD, President’s Malaria Initiative, Roll back malaria partnership, UNICEF, World Health Organization. Household survey indicators for malaria control; 2013.

[10] Vyas S, Kumaranayake L (2006). Constructing socio-economic status indices: how to use principal components analysis. Health Policy Plan.

[11] Zou G (2004). A modified poisson regression approach to prospective studies with binary data. Am J Epidemiol.

[12] Koenker H, Arnold F, Ba F, Cisse M, Diouf L, Eckert E (2018). Assessing whether universal coverage with insecticide-treated nets has been achieved: is the right indicator being used?. Malar J.

[13] Ministry of Health. The Uganda malaria reduction strategic plan 2014–2020. Kampala, Uganda; 2014.

[14] Rickard DG, Dudovitz RN, Wong MD, Jen HC, Osborn RD, Fernandez HE (2011). Closing the gap between insecticide treated net ownership and use for the prevention of malaria. Prog Community Health Partnersh.

[15] Deribew A, Birhanu Z, Sena L, Dejene T, Reda AA, Sudhakar M (2012). The effect of household heads training about the use of treated bed nets on the burden of malaria and anaemia in under-five children: a cluster randomized trial in Ethiopia. Malar J.

[16] Greenwood B, David P, Otoo-Forbes L, Allen S, Alonso P, Schellenberg JA (1995). Mortality and morbidity from malaria after stopping malaria chemoprophylaxis. Trans R Soc Trop Med Hyg.

[17] Taylor C, Florey L, Ye Y (2017). Equity trends in ownership of insecticide-treated nets in 19 sub-saharan african countries. Bull World Health Organ.

[18] Durrheim D, Govere J (2002). Malaria outbreak control in an african village by community application of ‘deet’mosquito repellent to ankles and feet. Med Vet Entomol.

[19] Rowland M, Freeman T, Downey G, Hadi A, Saeed M (2004). DEET mosquito repellent sold through social marketing provides personal protection against malaria in an area of all-night mosquito biting and partial coverage of insecticide‐treated nets: a case–control study of effectiveness. Trop Med Int Health.

[20] Deressa W, Yihdego YY, Kebede Z, Batisso E, Tekalegne A, Dagne GA (2014). Effect of combining mosquito repellent and insecticide treated net on malaria prevalence in Southern Ethiopia: a cluster-randomised trial. Parasit Vectors.

[21] Rowe A, Steketee R, Arnold F, Wardlaw T, Basu S, Bakyaita N (2007). Roll back malaria monitoring and evaluation reference group: viewpoint: evaluating the impact of malaria control efforts on mortality in sub-saharan Africa. Trop Med Int Health.

[22] Krezanoski PJ, Bangsberg DR, Tsai AC (2018). Quantifying bias in measuring insecticide-treated bednet use: meta-analysis of self-reported vs objectively measured adherence. J Glob Health.

